# The effects of antithrombotic therapy in *ab interno* trabeculotomy with a spatula-shaped microhook

**DOI:** 10.1371/journal.pone.0262548

**Published:** 2022-01-13

**Authors:** Satoru Kanda, Takashi Fujishiro, Takashi Omoto, Ryosuke Fujino, Kiyoshi Ishii, Makoto Aihara

**Affiliations:** 1 Department of Ophthalmology, Saitama Red Cross Hospital, Saitama-shi, Saitama, Japan; 2 Department of Ophthalmology, University of Tokyo Graduate School of Medicine, Bunkyo-ku, Tokyo, Japan; Cairo University Kasr Alainy Faculty of Medicine, EGYPT

## Abstract

To evaluate the effects of the discontinuation of antithrombotic drugs on intraocular pressure (IOP) reduction and complications from *ab interno* trabeculotomy for patients with glaucoma. We performed a retrospective chart review on the data of patients treated with antithrombotic agents who have undergone *ab interno* trabeculotomy through Tanito microhook combined with cataract surgery at the Asahi General Hospital and the Tokyo University Hospital, with 6 months of follow-up. The patients were classified into two groups depending on whether they discontinued (AT-) or continued (AT+) antithrombotic therapy during the perioperative phase. The demographics, pre- and postoperative IOP, medication score, best-corrected visual acuity (BCVA), and postoperative complications were analyzed preoperatively and postoperatively at 1 week and 1–6 months. The series included 44 eyes from 44 Japanese patients. The AT- and AT+ groups included 21 eyes from 21 patients and 23 eyes from 23 patients, respectively. The decrease in IOP from the baseline at 1 week postoperative was significantly different between the two groups (p = 0.009), but there were no significant differences observed in the other visits. Hyphema and IOP spikes exceeding 30 mmHg occurred in 10% and 10% of AT- participants, and in 43% and 26% of AT+ participants, respectively. Hyphema and spikes with hyphema occurred more frequently in the AT+ than in the AT- group (p = 0.02 and p = 0.05). The number of patients who had spikes was not significantly different (p = 0.27). In trabeculotomy using the Tanito microhook®, discontinuing antithrombotic therapy had better IOP-lowering effects and less postoperative complications.

## Introduction

Trabeculotomy aims to cleave the trabecular meshwork (TM) and the inner wall of the Schlemm’s canal (SC), which is the main site of resistance for aqueous outflow [1–7]. Conventional trabeculotomy is achieved via the *ab externo* approach through a conjunctival incision, which takes a relatively long operation time and induces conjunctival scarring, which may affect future filtration surgery. With the recent developments in new devices or implants, minimally invasive glaucoma surgery (MIGS) is emerging as a standard concept instead of *ab externo* trabeculotomy [8]. MIGS reduces the outflow resistance by an approach from the inside of the anterior chamber, involving only a small incision of the cornea. Thus, devices or implants for MIGS are minimally invasive and require high biocompatibility. Their safety and the fast recovery of patients in terms of visual acuity (VA) and quality of life (QOL) have been fully evaluated in outflow channel surgeries [1–3, 7–17].

The common MIGS procedures for the outflow pathway include incision of the trabecular tissue (e.g., Tanito *ab interno* microhook®: Inami & Co., Ltd, Tokyo, Japan) [1, 2, 7, 9, 14, 16], removal of the trabecular tissue (e.g., Trabectome^®^: NeoMedix Corporation, CA, USA; Kahook Dual Brade^®^: New World Medical, CA, USA) [1, 2, 10, 11, 15, 17], or implantation of a small device into the TM and SC (e.g., iStent, iStent inject^®^, and Hydrus MicroShunt) [12, 13, 17]. The clinical results of these devices have been reported in several studies [1, 2, 7, 9–17].

Trabeculotomy induces hyphema by causing blood to flow back from the episcleral vein through the collector channel after cleaving the TM. In cases of severe hyphema and intraocular pressure (IOP) elevation after surgery, washing the anterior chamber is sometimes necessary. Thus, discontinuation of antithrombotic therapy during the perioperative period should be considered, because antithrombotic therapy may affect the hyphema volume or washout. In trabeculotomy using the Tanito microhook®, there are no reports about the effect of antithrombotic therapy on perioperative complications. In *ab externo* trabeculotomy, there was a previous report about the effect of antithrombotic therapy [18]. In the previous report, the occurrence of hyphema was significantly different among those who continued and those that suspended antithrombotic therapy; however, the IOP 12 months after surgery did not differ. Furthermore, hyphema in the eyes of patients who continued antithrombotic therapy remained longer than in patients who suspended the therapy, and the former required irrigation of the anterior chamber more frequently. The reduction in IOP and MS 12 months after the surgery was comparable between these groups, but there are advantages in terms of safety and complications in the early postoperative phase after antithrombotic therapy discontinuation [18]. Furthermore, there have been no studies on the early postoperative phase in *ab interno* trabeculotomy, and a study around the perioperative period is desirable. Generally, antithrombotic therapy is discontinued in glaucoma surgery during the perioperative period. However, many patients cannot stop antithrombotic therapy when the risk of lethal systemic complications is considered. Therefore, our aim was to compare the surgical effectiveness and safety profile of *ab interno* trabeculotomy using the Tanito microhook® combined with cataract surgery among Japanese patients with open-angle glaucoma, who either continued or discontinued antithrombotic therapy.

## Methods

### Subjects

A consecutive series of *ab interno* trabeculotomy combined with cataract surgery cases between April 2018 and March 2019, performed by five surgeons at the Asahi General Hospital and Tokyo University Hospital with a minimum of 6 months of routine follow-up were included in this study. Eyes with past ophthalmic surgeries, such as trabeculotomy, trabeculectomy, goniosynechialysis, argon laser trabeculoplasty, selective laser trabeculoplasty, and vitrectomy, were excluded from the study. This observational study was approved by the Institutional Review Board of the Asahi General Hospital (registration number: 2019052116) and Tokyo University Hospital (registration number: 2217) and was conducted in accordance with the principles of the Declaration of Helsinki. All patients were treated with antithrombotic drugs, including warfarin potassium, aspirin, rivaroxaban, edoxaban tosilate hydrate, clopidogrel sulfate, apixaban, cilostazol, and prasugrel hydrochloride (Table 1). All antithrombotic therapy was by oral administration. The medical history of patients who received antithrombotic therapy is shown in Table 1. All patients read and signed the informed consent form before the surgery. The patients who have undergone trabeculotomy were classified into two groups: those who discontinued (AT-) and those who continued (AT+) antithrombotic therapy. In principle, antithrombotic therapy was discontinued in all patients who would be undergoing surgery. However, if the internal medicine department was unable to discontinue the medication due to the patient’s general condition before surgery, we decided to continue the medication and perform the surgery. Both groups had two patients who took Warfarin. Their prothrombin time international normalized ratio was around 2.0. However, their activated partial thromboplastin time and bleeding time were normal. Blood test of the other patients was normal. In comments of their physician, their medical management was appropriate and stable, and they had no underlying predisposition to bleeding. Warfarin potassium, aspirin, clopidogrel sulfate and prasugrel hydrochloride were discontinued 7 days prior to surgery. Rivaroxaban and edoxaban tosilate hydrate were discontinued 24 hours prior to surgery. In all cases, antithrombotic treatment was resumed the day after surgery. All patients simultaneously underwent cataract surgery via a temporal scleracorneal or corneal incision.

**Table 1:** Preoperative characteristics of patients who underwent trabeculotomy.

	AT-	AT+	P value
No. of eyes (patients)	21 (21)	23 (23)	
Age	76.5 ± 6.88	74.7 ± 5.87	0.38 *
Female / Male	7/14	11/12	
Right / Left	6/15	9/14	
Baseline			
IOP	18.6 ± 5.33	19.1 ± 6.68	0.79 *
Medication score	3.43 ± 1.68	3.35 ± 1.31	0.86 *
Log MAR VA	0.40 ± 0.42	0.28 ± 0.30	0.25 *
Indications			
POAG	17	18	1.0 **
SOAG	1	4	0.35 ***
PEG	3	1	0.33 ***
Antithrombotic drug			
warfarin	2	2	
aspirin	12	13	
ribaroxaban	3	3	
edoxaban	1	1	
clopidgrel	1	1	
apixaban	1	1	
cilostazol	1	1	
prasugrel	0	1	
Medical history			
Myocardial infarction	9	8	
stroke	8	8	
arrythmia	4	3	
Venous thrombosis	0	1	

IOP: intraocular pressure, Log MAR VA: logarithm minimal angle resolution visual acuity, POAG: primary open angle glaucoma, SOAG: secondary open angle glaucoma, PEG: pseudo exfoliation glaucoma, Data are presented as mean ± standard division or number.

* Calculated using linear model.

** Calculated using Pearson’s chi-square test.

*** Calculated using Fisher’s exact test.

### Surgical technique

All patients underwent surgery using a Tanito microhook®, which is a reusable hook with a spatula-shaped tip. This hook has three types: straight, angle-right, and angle-left, which allow surgeons to approach all quadrants of TM [14]. In *ab interno* trabeculotomy using a Tanito microhook®, two corneal ports were made at the 1–2 and 10–11 o’clock positions. After cataract surgery, the anterior chamber was filled with an ophthalmic viscosurgical device, either, Healon® (Abbott Medical Optics Inc., CA, USA), or Opegan-High® (Santen Pharmaceutical Co., Ltd., Japan). Then, the inner wall of the SC around the inferior 120–150° was cleaved with a Tanito microhook® through two ports, with assisted gonioscopy. Healon® and Opegan-High® were washed out using irrigation and aspiration. At end of surgery, the anterior chamber filled with balanced salt solution.

### Postoperative examination

A topical antibiotic (levofloxacin) and corticosteroid (betamethasone sodium phosphate) were postoperatively administered four times per day, plus a miotic agent (pilocarpine hydrochloride) three times per day, which were all tapered according to the postoperative course. IOP-lowering medications were restarted based on the surgeon’s decisions.

A retrospective chart review was conducted. Observational points were set at 1 week (within ± 2 days) and 1, 3, and 6 months (within ± 1 week) postoperatively. IOP was measured using Goldmann applanation tonometry. Baseline IOP was measured as the final IOP before surgery. Changes in IOP and medication scores (MS) over time were recorded. In the analysis of MS, combination drugs and oral acetazolamide administration were counted as 2 and 1, respectively. Postoperative routine use of miotic agents was not counted as IOP-lowering medication. The best-corrected visual acuity (BCVA) was measured using a decimal VA chart and was converted to the logarithm of the minimum angle of resolution (log MAR) VA. Changes in VA within 0.2 log MAR were considered stable.

### Statistical analyses

Preoperative and postoperative IOP was analyzed by Dunnett’s test. A linear model was used to analyze the decrease in IOP and MS between the two groups. Pearson’s Chi-square test and Fisher’s exact test were used to analyze the characteristics of patients and complications. Analyses were performed using R (R version 3.6.1; Foundation for Statistical Computing, Vienna, Austria). Statistical significance was set at p < 0.05.

## Results

The patient demographics of each group are presented in Table 1. A total of 44 eyes (15 right and 29 left eyes) of 44 patients with glaucoma (26 males and 18 females) were identified. The IOP and MS in the AT- and AT+ groups was 18.6 ± 6.88 mmHg and 3.43 ± 1.68 and 19.1 ± 6.68 mmHg and 3.35 ± 1.31, respectively. There was no significant difference between the two groups at the baseline.

Changes in IOP and MS are summarized in Tables 2 and 3. Comparing the two groups, differences in IOP and MS were observed one week after the operation (p = 0.009 and 0.0492, respectively). However, there was no difference observed in the remaining visits.

**Table 2:** Transition and comparison of IOP.

Time point	AT- (n = 21)		AT+ (n = 23)		P value **
	IOP at each time point (mmHg)	P value*	IOP at each time point (mmHg)	P value*	
Baseline	18.6 ± 5.33		19.1 ± 6.68		
1 week	14.3 ± 4.02	0.001	18.5 ± 5.55	0.98	0.009
1 month	12.9 ± 3.27	<0.001	14.9 ± 3.66	0.02	0.06
3 months	13.4 ± 2.66	<0.001	12.6 ± 3.99	<0.001	0.44
6 months	13.5 ± 2.20	<0.001	12.5 ± 3.91	<0.001	0.30

IOP: intraocular pressure, AT: antithrombotic therapy.

* Calculated using Dunnett’s test in IOPs between pre- and postoperative values.

** Calculated using linear model in reduction number and reduction rate between AT- and AT+.

**Table 3:** Transition of medication score.

Time point	AT- (n = 21)		AT+ (n = 23)		P value **
	MS at each time point (mmHg)	P value*	MS at each time point (mmHg)	P value*	
Baseline	3.43 ± 1.68		3.35 ± 1.31		
1 week	0.048 ± 0.21	<0.001	0.43 ± 0.88	<0.001	0.0492
1 month	0.43 ± 0.90	<0.001	0.61 ± 0.87	<0.001	0.52
3 months	0.52 ± 0.91	<0.001	0.70 ± 0.91	<0.001	0.54
6 months	0.57 ± 0.95	<0.001	0.71 ± 0.93	<0.001	0.67

MS: medication score, AT: antithrombotic therapy.

* Calculated using Dunnett’s test in MS between pre- and postoperative values.

** Calculated using linear model in MS between AT- and AT+.

Changes in the log MAR VA are summarized in Table 4. No significant differences were observed in all visits.

**Table 4:** Transition of visual acuity.

Time point	AT- (n = 21)		AT+ (n = 23)		P value **
	Log MAR VA at each time point	P value*	Log MAR VA at each time point	P value*	
Baseline	0.41 ± 0.42		0.28 ± 0.30		
1 week	0.13 ± 0.36	0.05	0.23 ± 0.64	0.98	0.56
1 month	0.13 ± 0.31	0.05	0.066 ± 0.27	0.21	0.50
3 months	0.11 ± 0.34	0.03	0.061 ± 0.28	0.20	0.64
6 months	0.093 ± 0.34	0.02	0.056 ± 0.28	0.18	0.70

Log MAR VA: logarithm minimal angle resolution, AT: antithrombotic therapy.

* Calculated using Dunnett’s test in IOPs between pre- and postoperative values.

** Calculated using linear model in Log MAR VA between AT- and AT+.

We also analyzed the safety profile of trabeculotomy and summarized the results in Table 5. Hyphema with niveau formation (hyphema) and transient IOP spike exceeding 30 mmHg were two common postoperative complications. Table 5 shows that hyphema and spikes with hyphema were significantly more frequent in the AT+ than the AT- group (P = 0.02, and 0.0493, respectively). As for the level of niveau, there was no significant difference in the number of cases in which the height of the niveau rested on the pupil. (p = 0.66) Hyphema was observed intraoperatively among all eyes, but no eye required postoperative washout. There was no significant difference in the duration of hyphema between the two groups. (p = 0.82) The duration of hyphema was from the day after the surgery day to the day when the absence of hyphema was confirmed.

**Table 5:** Comparison of complication.

		AT-	AT+	P value *
Hyphema with niveau formation		2	10	0.02
transient IOP spike		2	6	0.27
spike and hyphema		0	5	0.0493
worsening of VA		1	1	1.00

IOP: intraocular pressure, VA: visual acuity, AT: antithrombotic therapy.

* Calculated using Fisher’s exact test in complications between AT- and AT+.

All patients were treated with IOP-lowering medications, and no patient needed additional trabeculectomy during the observational period. There was no exacerbation of systemic disease due to the withdrawal of antithrombotic therapy 6 months after the surgery. There was one case of worsened VA in the AT+ (4.8%) and in the AT- (4.3%) group. Both cases had an impaired central visual field that was maintained before surgery. Furthermore, both cases had hyphema with a spike.

## Discussion

Conventional trabeculotomy, which is performed using an *ab externo* approach through metal trabecular probes, requires a relatively long operation time and induces conjunctival scarring, which may affect future filtration surgery. By contrast, newer techniques performed using an *ab interno* approach require only minimal invasion. Therefore, MIGS is emerging as a standard concept in the field [8]. In the current study, we evaluated the effects of antithrombotic therapy during the perioperative phase. We found that IOP one week after surgery was significantly lower in the AT- than in the AT+ group. This implied that suspending antithrombotic therapy was more effective in immediately decreasing IOP and avoiding IOP elevation after surgery. In general, many patients are affected by their central visual field. These patients may be suited to antithrombotic therapy discontinuation because among them, it is desirable to immediately decrease IOP and avoid IOP elevation.

In the current study, the occurrence of hyphema and spike with hyphema was more frequent in the AT+ than in the AT- group. For this reason, blood clots and red blood cells (RBCs) may block the flow from the anterior chamber to the episcleral vein through the TM or collector channel. The relationship between IOP elevations similar to spikes and RBCs similar to hyphema had been discussed [19–21]. These reports suggested the possibility that IOP elevation occurred because degenerated RBCs blocked the flow at TM. Xu et al. showed that the mean IOP elevation at 48 hours after hemorrhage was 46.5 mmHg [21]. Our cases may also have a spike and an IOP elevation with a mechanism similar to that in previous reports.

In almost all cases, hyphema occurred during the surgery and disappeared gradually within 1–2 weeks postoperatively. In some cases, IOP decreased with a decrease in hyphema formation. Nevertheless, hyphema forms niveau and fluttering in the anterior chamber as single RBCs. We counted niveau formation as hyphema and did not mention the fluttering type because of quantifying difficulties, similar to previous studies [9, 18]. However, the fluttering type hyphema also seems to have influenced IOP. Therefore, a discussion and examination of the method to quantify hyphema are necessary. Furthermore, the relationship between the amount of hyphema and IOP should be studied in the future.

In the current study, there were two cases of worsened VA. The postoperative visual field test showed central visual field impairment that was not observed preoperatively; therefore, the worse VA was thought to be caused by progression of ​visual field impairment. Furthermore, spikes with hyphema occurred in both cases. In cases where central vision is imminently impaired, the central vision may be affected by postoperative IOP elevation, and it may be better to discontinue antithrombotic therapy to decrease the possibility of postoperative IOP elevation.

People who undergo glaucoma surgery tend to be advanced in age and are taking some types of antithrombotic therapy. We stopped antithrombotic therapy mainly to lower the bleeding risk in both groups in our study. However, an increase in the number of elderly patients who must continue antithrombotic therapy is inevitable. According to our findings, the AT- group had better IOP in the early operative phase and lower frequency of complications than the AT+ group. Thus, we believe that discontinuation of antithrombotic therapy is a tolerable method for patients who can stop this therapy.

This study has some limitations. This was a non-randomized retrospective case series with a short follow-up period. Thus, further studies are needed to evaluate the long-term clinical results and safety profiles after trabeculotomy. Nevertheless, the current study provides empirical support for the decision regarding the proper indications for trabeculotomy.

In conclusion, we need to consider the discontinuation of antithrombotic therapy comprehensively, taking into account the patient’s general condition and visual field if the emphasis is on early postoperative IOP.
